# A Comprehensive Analysis of the Genetic Diversity of *Plasmodium falciparum* Histidine-Rich Protein 2 (PfHRP2) in the Brazilian Amazon

**DOI:** 10.3389/fcimb.2021.742681

**Published:** 2021-09-21

**Authors:** Gabriel Luíz Costa, Maria Eduarda Pereira Mascarenhas, Thamires Oliveira Gasquez Martin, Laura Guimarães Fortini, Jaime Louzada, Dhélio Batista Pereira, Anna Caroline Campos Aguiar, Luzia Helena Carvalho, Cristiana Ferreira Alves de Brito, Cor Jesus Fernandes Fontes, Tais Nóbrega de Sousa

**Affiliations:** ^1^Molecular Biology and Malaria Immunology Research Group, Instituto René Rachou, Fundação Oswaldo Cruz (FIOCRUZ), Belo Horizonte, Brazil; ^2^Hospital Universitário Júlio Müller, Federal University of Mato Grosso, Cuiabá, Brazil; ^3^Federal University of Roraima, Boa Vista, Brazil; ^4^Center for Research in Tropical Medicine (Cepem), Porto Velho, Brazil; ^5^Bioscience Department, University of São Paulo, Santos, Brazil

**Keywords:** malaria, Plasmodium falciparum, pfhrp2 deletion, rapid diagnostic test (RDT), genetic diversity

## Abstract

Early diagnosis and treatment are fundamental to the control and elimination of malaria. In many endemic areas, routine diagnosis is primarily performed microscopically, although rapid diagnostic tests (RDTs) provide a useful point-of-care tool. Most of the commercially available RDTs detect histidine-rich protein 2 (HRP2) of *Plasmodium falciparum* in the blood of infected individuals. Nonetheless, parasite isolates lacking the *pfhrp2* gene are relatively frequent in some endemic regions, thereby hampering the diagnosis of malaria using HRP2-based RDTs. To track the efficacy of RDTs in areas of the Brazilian Amazon, we assessed *pfhrp2* deletions in 132 P*. falciparum* samples collected from four malaria-endemic states in Brazil. Our findings show low to moderate levels of *pfhrp2* deletion in different regions of the Brazilian Amazon. Overall, during the period covered by this study (2002-2020), we found that 10% of the *P. falciparum* isolates were characterized by a *pfhrp2* deletion. Notably, however, the presence of *pfhrp2*-negative isolates has not been translated into a reduction in RDT efficacy, which in part may be explained by the presence of polyclonal infections. A further important finding was the discrepancy in the proportion of *pfhrp2* deletions detected using two assessed protocols (conventional PCR *versus* nested PCR), which reinforces the need to perform a carefully planned laboratory workflow to assess gene deletion. This is the first study to perform a comprehensive analysis of PfHRP2 sequence diversity in Brazilian isolates of *P. falciparum*. We identified 10 PfHRP2 sequence patterns, which were found to be exclusive of each of the assessed regions. Despite the small number of PfHRP2 sequences available from South America, we found that the PfHRP2 sequences identified in Brazil and neighboring French Guiana show similar sequence patterns. Our findings highlight the importance of continuously monitoring the occurrence and spread of parasites with *pfrhp2* deletions, while also taking into account the limitations of PCR-based testing methods associated with accuracy and the complexity of infections.

## Introduction

Between 2000 and 2019, malaria-related deaths worldwide were reduced by 44%, from 736,000 in 2000 to 409,000 in 2019 (World Health Organization, 2020). Nevertheless, the spread of the COVID-19 pandemic to all malaria-endemic countries was responsible for the disruption of essential malaria services (World Health Organization, 2020). This has compromised the WHO global strategy, which had been designed to reduce the incidence of malaria and mortality rates by at least 90% by 2030 (World Health Organization, 2015). In 2020, Brazil reported approximately 141,000 cases of malaria (84% caused by *Plasmodium vivax* and 16% by *Plasmodium falciparum*) (BRASIL. Ministério da Saúde, 2021). Although these numbers represent a reduction in the total number of cases of *P. vivax*, there was a 32.6% increase in *P. falciparum* cases when compared with 2019.

Early diagnosis and treatment are fundamental components of strategies designed to control and eliminate malaria. In many endemic areas, the routine diagnosis is primarily performed microscopically. Nonetheless, the rapid diagnostic tests (RDTs) provide a useful tool when microscopy facilities are unavailable, particularly in remote areas and in the absence of a well-trained professionals (BRASIL. Ministério da Saúde, 2009). RDTs can be used to detect specific *Plasmodium* antigens in the blood stream, such as histidine-rich protein 2 (HRP2) and lactate dehydrogenase (LDH) (World Health Organization, 2016). The sensitivity of RDTs is dependent on detection of the target protein; however, since the 2000s, isolates with deletion of the gene *pfhrp2* gene have been reported in Africa and South America (Baker et al., 2005; Baker et al., 2010; Gamboa et al., 2010; Houzé et al., 2011). In Brazil, *pfhrp2* deletions have been reported in some endemic states, such as Acre, Rondônia, and Amazonas (Rachid Viana et al., 2017; Góes et al., 2021).

PfHRP2 comprises different types of histidine and alanine repeats (Rock et al., 1987). An extensive study conducted by the Malaria RDT Quality Assurance Program reported a high variability in the *pfhrp2* sequence of isolates from Asia and the southwest Pacific (Baker et al., 2005; Lee et al., 2006), and it was subsequently proposed that certain types of alanine/histidine repeats were associated with variations in the sensitivity of RDTs, interfering with the test efficacy (Baker et al., 2005; Lee et al., 2006). However, other studies have shown contrasting data concerning the effect of PfHRP2 variability on RDT efficacy (Baker et al., 2010; Kumar et al., 2012; Atroosh et al., 2015; Willie et al., 2018).

In recent years, reports of *pfhrp2* deletions have increased, including 16 reports from 15 endemic countries only between 2019 and 2020 (World Health Organization, 2020). The WHO is currently tracking these publications to monitor *pfhrp2* deletions and recommends that countries conduct representative baseline surveys among suspected malaria cases with false-negative RDT results (World Health Organization, 2020). In this study, we evaluated the proportion of *pfhrp2* deletion in *P. falciparum* isolates from four malaria-endemic states of the Brazilian Amazon (Amapá, Mato Grosso, Rondônia and Roraima state) over a period of 18 years (2002 – 2020). RDT results were interpreted considering the *pfhrp2* profile, parasitemia, and complexity of infection. Additionally, a comprehensive analysis of PfHRP2 sequence variability was conducted for parasite isolates from South America.

## Materials and Methods

### Study Population and Parasite Isolates

In this study, we assessed *P. falciparum* samples collected from four Brazilian states over differing periods of time: 11 samples from Macapá (Amapá state, AP) in 2004; 40 from Cuiabá (Mato Grosso state, MT) between 2002 and 2012; 33 from Porto Velho (Rondônia state, RO) between 2008 and 2018; and 48 from Boa Vista (Roraima state, RR) between 2018 and 2020 (**Table 1**). Among these locations, Cuiabá/MT lies within a region in which malaria is in the pre-elimination phase, and thus most individuals were infected elsewhere (mainly in Rondônia and Pará states). The patients included in this study were symptomatic and had sought treatment provided by the local public health services. *Plasmodium* spp. infection was confirmed by microscopy based on Giemsa-stained thick blood smears evaluated by well-trained microscopists in accordance with the malaria diagnosis guidelines of the Brazilian Ministry of Health. Samples collected in Rondônia and Roraima states were tested using the SD Bioline Malaria Ag P.f/Pan test (Abbott, Inc.; Korea) according to the manufacturer’s instructions. Samples of dried blood from Mato Grosso were tested using SD Bioline Malaria Ag P.f (Standard Diagnostics, Inc.; Korea).

**Table 1:** Description of 132 *Plasmodium falciparum*-infected patients enrolled in this study, *pfhrp2* deletion profile and RDT result.

Site	N	Year of sample collection	N° isolates *pfhrp2-negative*	N° RDT-negative^a^ (Total)	Male gender^b^ (%)	Age^b^, mean (SD)	Parasitemia^c^, geometric mean (95% CI)
Amapá	11	2004-2005	0	0 (3)	81.8	32 (11)	1,561 (781 – 3,119)
Mato Grosso	40	2002-2012	4	0 (23)	86.8	35 (14)	1,848 (924 – 3,694)
	28	2002-2007	4	0 (11)			
	12	2008-2012	0	0 (12)			
Rondônia	33	2008-2018	2	2 (25)	75.0	38 (11)	1,486 (766 – 2,881)
	7	2008	0	NA			
	26	2017-2018	2	2 (25)			
Roraima	48	2018-2020	7	2 (43)	72.9	35 (11)	1,545 (861 – 2,770)
Total	132	2002-2020	13	4 (94)	78.3	36 (12)	1,617 (1,151 – 2,273)

NA, Not available; SD, Standard deviation.

a PfHRP2-based RDT.

b Data calculated using all samples from the region.

c Parasites/µL.

### Confirmation of *P. falciparum* Infection by Quantitative PCR (qPCR)

Genomic DNA was extracted from 300 µL of whole blood samples collected in EDTA using a Gentra Puregene blood kit (Qiagen, Minneapolis, MN, USA) according to the manufacturer’s instructions and eluted in 50 µL of DNA hydration solution. Molecular diagnosis was performed for all samples to confirm the *P. falciparum* infection. Species-specific quantitative PCR assays targeting a multi-copy target (non-ribosomal Pvr47/Pfr364 sequences) were carried out as previously described (Amaral et al., 2019).

### Characterization of the *pfhrp2* Gene

To assess *pfhrp2* (PF3D7_0831800) deletion, we amplified exon 2 of this gene using the primers described by Baker et al. (2005). In addition, the amplification of the two *pfhrp2* flanking genes, MAL7P1.230 (PF3D7_0831900) and MAL7P1.228 (PF3D7_0831700), was performed as described by Gamboa et al. (2010). *pfhrp2* and the flanking genes were amplified using single-round PCR protocols. To confirm the results obtained, a nested PCR was performed on the same samples (Abdallah et al., 2015). This protocol amplifies the exon 1, the intron, and exon 2 of *pfhrp2*. The amplification of MAL7P1.230 and MAL7P1.228 was also assessed by the nested PCR, as described by Abdallah et al. (2015). The primers and PCR conditions used for amplification are listed in **Supplementary Table 1**. All samples with negative reactions were re-amplified to confirm the gene deletion. The results of both protocols were compiled to characterize the deletion profiles of the analyzed samples. Thus, when one of the protocols yielded a positive reaction, the presence of the gene was reported independently of the protocol applied. Given the low amounts of DNA for many of the samples, only *pfhrp2*-negative samples were subjected to PCR to assess the presence of *pfhrp3* (PF3D7_1372200) and its flanking genes, MAL13P1_475 (PF3D7_1372100) and MAL12P1_485 (PF3D7_1372400). Amplification of *pfhrp3* and the two flanking genes was performed using the single-round PCR protocols described by Baker et al. (2005) and Gamboa et al. (2010), respectively. The laboratory line 3D7 was used as a positive control for the PCR analyses of *pfhrp2*, *pfhrp3*, and their respective flanking genes.

### DNA Sequencing and PfHRP2 Sequence Analysis

The primers described by Baker et al. (2005) were used for *pfhrp2* sequencing (**Supplementary Table 1**). PCR products were purified using a QIAquick PCR Purification Kit (QIAGEN, Chatsworth, CA, USA) and then sequenced using an ABI 3730xL DNA analyzer (Thermo Fisher Scientific, Waltham, MA, USA). All samples were sequenced at least twice using forward and reverse primers. Nucleotide sequences were aligned and translated into amino acids using the Mega X software (Kumar et al., 2018). The amino acid repeat types were identified using a numeric code according to Baker et al. (2005; 2010). We extended the variability analysis to samples from other South American countries, examining sequences from Peru (accession numbers AY816272; FJ871164; FJ871352, and FJ871353) (Baker et al., 2005; Baker et al., 2010), Suriname (accession number FJ871350) (Baker et al., 2010), Colombia (accession numbers FJ871211 and FJ871254) (Baker et al., 2010), French Guiana (accession numbers KC558574 and KC558602) (Trouvay et al., 2013) and Brazil (accession numbers FJ871160, FJ871209, FJ871210, AY816292-AY816295, and AY816240) (Baker et al., 2005; Baker et al., 2010).

### Analysis of *P. falciparum* Population Diversity

To determine whether the infections were mono- or polyclonal, three microsatellites were genotyped using the ABI 3730xL DNA analyzer, namely, *Polyα*, *PfPK2*, and *TA87* (Su and Wellems, 1996). The primers used in the PCR protocol are listed in **Supplementary Table 1**, and the electrophoresis products were analyzed using GeneMapper™ software version 4.1 (Thermo Fisher Scientific, Waltham, MA, USA). For electropherogram analysis, the minimum peak height was set to 200 arbitrary fluorescence units. Additionally, to exclude artifact peaks, we used a cut-off value for minor peak detection of one-third the height of the predominant peak. Population genetics analyses were performed based on the predominant allele at each locus (i.e., that showing the highest electrophoretic peak). The expected heterozygosity (*H*_E_), mean number of alleles per locus, and allele frequencies were calculated using Arlequin v.3.5 software (Excoffier et al., 2007). The multiplicity of infection (MOI) was calculated by dividing the total number of alleles by the number of samples for each marker.

## Results

### Deletion Profile of *pfhrp2* and Its Flanking Genes

In this study, we analyzed *pfhrp2* deletion profiles in 132 samples collected from individuals in four states within the Brazilian Amazon, who were established to have clinical malaria caused by *P. falciparum*. All subjects were diagnosed with *P. falciparum* infection at enrollment based on a microscopic examination of samples, with subsequent confirmation by molecular diagnosis. Most patients were men with a mean age of 36 years (SD = 12 years) (**Table 1**).

**Figure 1:**
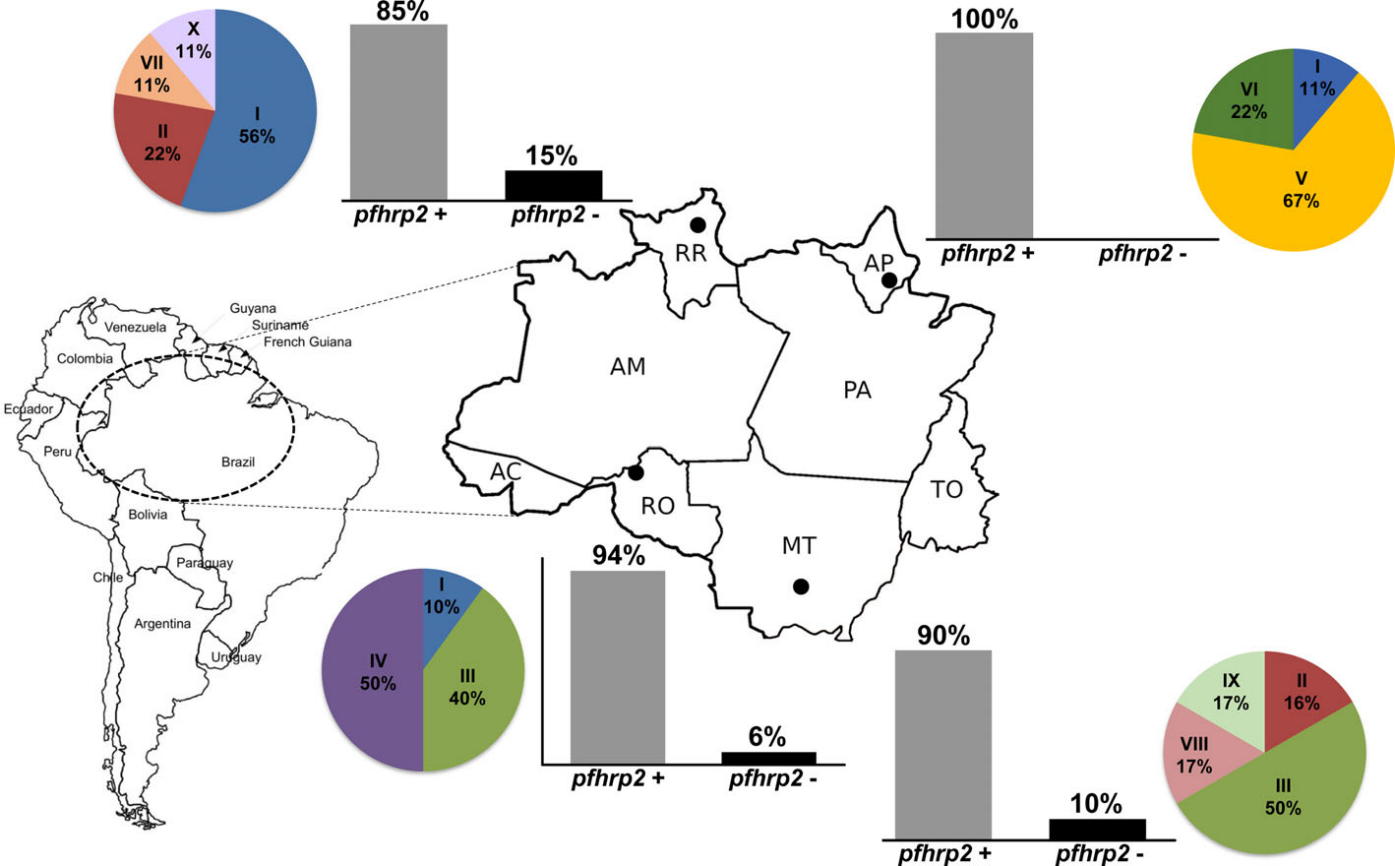
The deletion profiles and variability patterns of *pfhrp2* sequences of *Plasmodium falciparum* isolates from regions in the Brazilian Amazon. Bar plots show the proportion of *pfhrp2* deletion in each of the surveyed states, and the pie charts show the frequency distributions for the patterns of *pfhrp2* sequences. The localities of sample collection are shown on the map: Macapá, Amapá state (AP); Cuiabá, Mato Grosso state (MT); Porto Velho, Rondônia state (RO); Boa Vista, Roraima state (RR).

We analyzed the presence of the *pfhrp2* gene using two PCR protocols, namely, conventional and nested PCRs. Only samples that were successfully amplified for molecular diagnosis were included in this analysis. The results obtained using both protocols were compiled to characterize the deletion profiles of the analyzed samples. The results revealed that none of the samples obtained from individuals in Amapá state (n = 11) had a *pfhrp2* deletion (**Figure 1** and **Table 1**), whereas 6% (2 of 33) of the samples collected from Rondônia were found to be *pfhrp2*-negative. However, higher frequencies of *pfhrp2* deletion were observed among the samples collected in Roraima (14.6%, 7 of 48) and Mato Grosso (10%, 4 of 40). All samples from Mato Grosso with *pfhrp2* deletion were collected prior to 2007 (**Table 1**), whereas in contrast, in Rondônia and Roraima, gene deletion was detected in samples collected more recently. Considering all samples assayed in this study, the *pfhrp2* deletion rate reached 10% (13 of 132), and notably, approximately the same proportion of deletions (10%) was detected for samples collected in each of the two periods of survey, before and after 2008. Furthermore, we detected several types of deletion profile based on analyses of *pfhrp2* and the two flanking genes (**Table 2**), with the upstream gene MAL7P1.230 being the most frequently absent.

**Table 2:** Distinct profile of *pfhrp2* deletions found in the samples analyzed.

MAL7P1_230	*pfhrp2*	MALP1_228	N (%)^a^
Present	Present	Present	69 (51.5%)
Absent	Present	Present	45 (33.6%)
Absent	Present	Absent	3 (2.2%)
Present	Present	Absent	2 (1.5%)
Absent	Absent	Absent	**6 (4.5%)**
Absent	Absent	Present	**5 (3.7%)**
Present	Absent	Absent	**2 (1.5%)**

a Deletion profiles are in bold.

**Figure 2:**
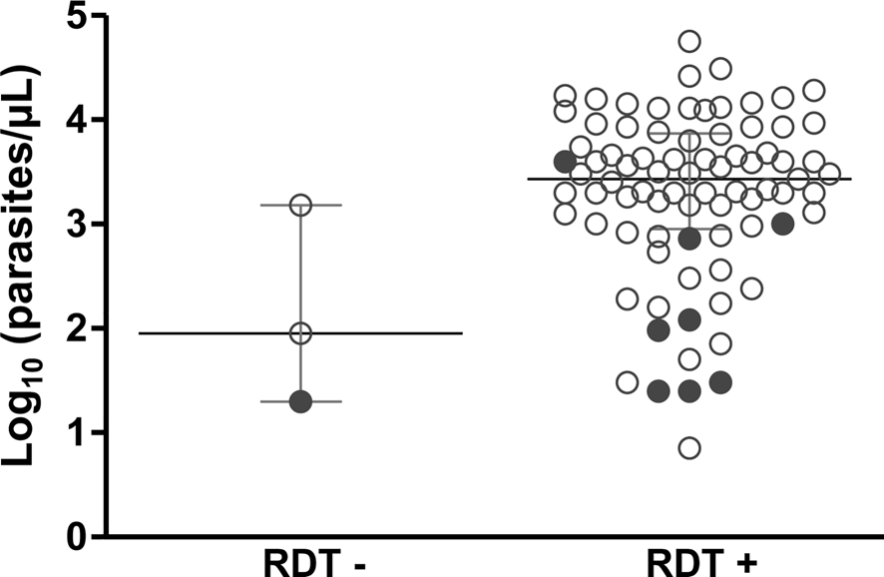
Association between the results of rapid diagnostic tests (RDTs) of malaria and parasitemia for subjects with clinical malaria caused by *P. falciparum*. Parasitemia (parasites/µL) in 84 samples was estimated based on microscopic analysis. Samples from Rondônia and Roraima were screened using an SD Bioline Malaria Ag P.f/Pan test (Abbott) at enrollment; samples of dried blood from Mato Grosso were tested using SD Bioline Malaria Ag P.f (Standard Diagnostics). *pfhrp2*-negative samples are shown in solid circle. RDT - and RDT + indicate negative and positive RDT results, respectively.

In total, 94 (71%) samples were tested using RDT, only four (4.3%) of which were negative for *P. falciparum* infection (**Table 1**). One of the 94 samples (1.1%) detected negative based on RDT and had a *pfhrp2* deletion. Nonetheless, this sample also had a very low parasitemia (20 parasites/µL) (**Figure 2**).

For the 84 samples with available parasitemia counts, most RDT-negative samples had low parasitemia (range = 20–1,500 parasites/µL) (**Figure 2**). Of note, eight samples characterized as *pfhrp2*-negative were detected by RDT (**Figure 2** and **Supplementary Table 2**). Preliminary analysis based on conventional PCR revealed that these samples were *pfhrp3*-negative. RDT data were unavailable for the remaining four *pfhrp2-*negative samples.

### Comparison of the *pfhrp2* Deletion Profiles Determined Using Different Protocols

**Figure 3:**
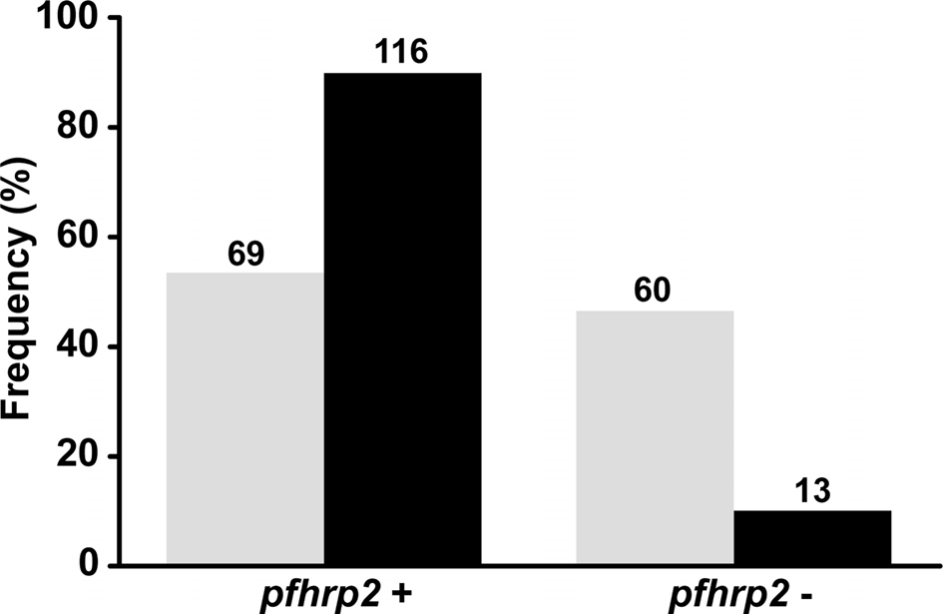
Comparison between protocols used in this study for the assessment of *pfhrp2* and flanking gene deletions. Absolute values are shown on bars. Gray: results of *pfhrp2* deletion based on Protocol 1 (conventional PCR using the primers described by Baker et al., 2005 and Gamboa et al., 2010); black: results of *pfhrp2* deletion based on Protocol 2 (nested PCR using the primers described in Abdallah et al., 2015). N = 129.

Initially, we analyzed the *pfhrp2* and flanking genes using conventional PCR (Protocol 1) with previously described primers (Baker et al., 2005; Baker et al., 2010). The results revealed that a large proportion of the isolates were characterized by complete deletions (**Figure 3**). To confirm these findings, we reanalyzed the same samples using the nested PCR protocol (Protocol 2) described by Abdallah et al. (2015). A comparison of the two protocols revealed a significant difference in the detected *pfhrp2* deletion rates (46% for Protocol 1 *vs.* 10% for Protocol 2). Moreover, there was essentially no reproducibility between the protocols for samples with low parasitemia (geometric mean = 1,795 parasites/µL, 95% CI = 1,131–2,847 for samples with concordant results *vs.* geometric mean = 1,304, 95% CI = 776–2,190 for samples with discordant results, *P* = 0.166, as determined using the Mann-Whitney test).

### Genetic Diversity of *P. falciparum* Samples

The genetic diversity among geographically discrete *P. falciparum* populations was characterized by genotyping three polymorphic microsatellites (*TA87*, *Polyα*, and *PfPK2*). Owing to insufficient amounts of sample DNA to perform all molecular assays, only 99 (75%) of the total 132 samples were successfully genotyped (the allele frequencies are given in **Supplementary Table 2**). We accordingly established that most infections were polyclonal (62 of 99), with multiplicity of infection (MOI) varying from 1.06 to 1.66 (**Table 3**). Overall, the parasite population studied was found to be highly diverse, with a total heterozygosity index of 0.708 (SD = 0.093). Among the four populations, the genetic diversity was observed to be higher in the states of Roraima and Rondônia, as indicated by the mean number of alleles per locus and heterozygosity indices (**Table 3**). In contrast, *P. falciparum* populations from Mato Grosso state were characterized by low genetic diversity with a lower number of alleles per locus and heterozygosity index.

**Table 3:** Proportion of multiplicity of infection and genetic diversity of *Plasmodium falciparum* populations from 4 endemic areas of the Brazilian Amazon.

Population	No. of isolates	MOI	No. of alleles per locus, mean ± SD	Within-population Diversity (*H*_E_), mean ± SD
Amapá	11	1.06	2.6 ± 1.1	0.527 ± 0.191
Mato Grosso	9	1.66	2.0 ± 1.7	0.250 ± 0.433
Rondônia	33	1.20	6.0 ± 2.0	0.636 ± 0.192
Roraima	46	1.27	5.3 ± 2.5	0.595 ± 0.111

Ten of the 13 samples with a *pfhrp2* gene deletion were successfully genotyped, and all but one sample was characterized by a polyclonal infection (**Supplementary Table 3**). Among these 10 samples, nine were tested using RDT, and only a single sample with very low parasitemia (20 parasites/µL) was found to be negative. Notably, the monoclonal infection was positive for RDT.

### HRP2 Sequence Variability

Overall, the *pfhrp2* gene was successfully amplified from 34 samples and thereafter subjected to amino acid repeat type analysis as described by Baker et al. (2005; 2010). Notably, amino acid repeat compositions were found to be similar for all regions analyzed (**Table 4**), with the sequences of all samples starting with a type 1 repeat (AHHAHHVAD) and ending with a type 12 repeat (AHHAAAHHEAATH). Repeat types 2 (AHHAHHAAD) and 7 (AHHAAD) were shown to have the highest number of repeat units per sample, whereas other repeat types were rarely present, such as type 4 (AHH), which was observed only in Mato Grosso samples. Repeat types 9 (AAY), 11 (AHN), 13 (AHHASD), and 14 (AHHAHHATD) were absent from all regions.

**Table 4:** Repeat types and number of repeat units in *pfhrp2* gene for 34 *Plasmodium falciparum* isolates from the Brazilian Amazon region.

Region	Minimum and maximum number of repeat units^a^													
	1	2	3	4	5	6	7	8	9	10	11	12	13	14
Amapá (N = 9)	4-6	9	1-2	0	2	3-4	6-10	1	0	2-3	0	1	0	0
Mato Grosso (N = 6)	2-4	9-12	2	0-1	0-2	3-6	8-11	0-1	0	0-3	0	1	0	0
Rondônia (N = 10)	2-4	9-12	1-2	0	1-2	2-4	8-9	1	0	0-2	0	1	0	0
Roraima (N = 9)	2-4	9-12	2	0	0-2	3-5	8-11	0-1	0	0-3	0	1	0	0

^a^Amino acid repeat types were identified as previous described (Baker et al., 2005; Baker et al., 2010).

**Figure 4:**
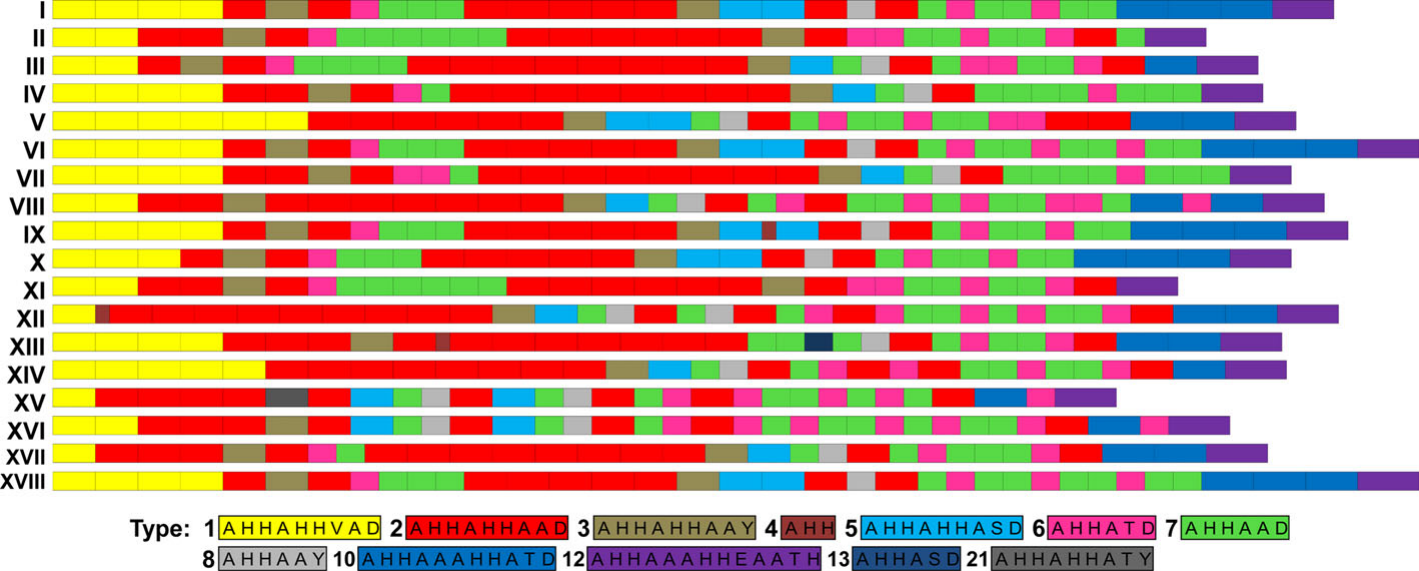
Patterns of PfHRP2 sequences of *P. falciparum* isolates from South America. Amino acid repeat types were identified as previously described (Baker et al., 2005; Baker et al., 2010). PfHRP2 sequence patterns I to X were identified in isolates sequenced in this study.

On the basis of the types and numbers of repeat units present in PfHRP2, we defined 10 distinct sequence patterns (patterns I to X in **Figure 4**) in the *P. falciparum* isolates sequenced in this study (**Figure 1**). The predominant pattern tended to differ among regions (**Figure 1**), with only Rondônia and Mato Grosso states having a common sequence (pattern III) at high frequency, which was present in samples collected during the same period from 2006 to 2008.

By analyzing the PfHRP2 sequences of isolates from other South America countries, we found pattern I to be the most prevalent pattern in Brazil and French Guiana (**Table 5**). However, the lack of available data precludes us from drawing conclusions regarding the diversity of PfHRP2 sequences in other countries.

**Table 5:** Frequencies of PfHRP2 sequence patterns of *Plasmodium falciparum* isolates from South America.

Patterns	Frequency (%)				
	Brazil	French Guiana	Colombia	Peru	Suriname
I	11 (26.2)	16 (55.2)	0	0	0
II	3 (7.1)	0	0	0	0
III	8 (19.0)	0	0	1 (25.0)	0
IV	6 (14.3)	0	0	0	0
V	6 (14.3)	4 (13.8)	0	0	0
VI	2 (4.8)	3 (10.3)	0	0	0
VII	1 (2.4)	0	0	0	0
VIII	1 (2.4)	1 (3.5)	0	0	0
IX	1 (2.4)	0	0	0	0
X	1 (2.4)	0	0	0	0
XI	1 (2.4)	1 (3.5)	0	0	0
XII	1 (2.4)	0	0	0	1 (100.0)
XIII	0	0	0	2 (50.0)	0
XIV	0	0	0	1 (25.0)	0
XV	0	0	1 (50.0)	0	0
XVI	0	0	1 (50.0)	0	0
XVII	0	3 (10.3)	0	0	0
XVIII	0	1 (3.5)	0	0	0
**Total**	**42**	**29**	**2**	**4**	**1**

## Discussion

Deletion of *pfhrp2/3* in the *P. falciparum* genome raises concerns regarding the efficacy of RDTs, given that parasites characterized by such deletions may not be identified by RDTs that are based on the detection of histidine-rich protein 2 (HRP2). According to WHO guidelines, the threshold for RDT change is a *pfhrp2* deletion rate greater than 5% causing false-negative RDT results (World Health Organization, 2020). In the present study, we assessed the *pfhrp2* deletion profile of 132 samples from four different states of the Brazilian Amazon. Our findings revealed that although the deletion of this gene is present in these regions, the proportion of false-negative RDT results attributable to *pfhrp2* deletion appears to be very low (1%). These findings can be attributed to polyclonal infections, owing to the presence of genetically distinct parasites differing in PfHRP2 expression that infect the same host. However, at this time we cannot exclude the possibility of cross-reactivity with PfHRP3.

Overall, 10% of the *P. falciparum* isolates analyzed were found to have a *pfhrp2* deletion, which was confirmed by the finding that most isolates were also frequently characterized by deletions of the genes flanking *pfhrp2*. Isolates in samples collected from Roraima were found to have the highest proportion (15%) of *pfhrp2* gene deletion, followed by the Mato Grosso (10%), and Rondônia (6%) isolates. These findings contrast with previous observations in other areas of the Brazilian Amazon, with rates of 32% to 72% and 54% to 100% being reported in the states of Acre and Amazonas, respectively (Rachid Viana et al., 2017; Góes et al., 2021). More consistent with our present findings, Rachid Viana et al. (2017) reported a low proportion (3%) of *pfhrp2* deletions in Rondônia state for isolates sampled in the Monte Negro municipality. We and others have also found that the MAL7P1_230 gene flanking *pfhrp2*, which encodes a *Plasmodium* exported protein (PHIST) of unknown function (https://plasmodb.org/), is frequently deleted in Brazilian samples (range = 21%–45%) (Rachid Viana et al., 2017), whereas the other flanking gene, MAL7P1_228, encoding for a putative heat shock 70 protein, was less frequently deleted (range = 0.5%–11%) in the same samples.

The present study is the first to assess the deletion profile of *pfhrp2* in isolates from Amapá state, and although we were only able to analyze a relatively few samples, none of these showed deletions of this gene. Consistently, previous studies in the neighboring Brazilian state of Pará and French Guiana have reported the absence of *pfhrp2* deletions in isolates obtained from both areas (Trouvay et al., 2013; Rachid Viana et al., 2017). In contrast, as previously mentioned, we detected a high proportion of *pfhrp2*-negative isolates (15%) from Roraima state, which borders Guyana and Venezuela, and notably, *P. falciparum* samples collected in Roraima were mainly obtained from travelers or workers originating from Venezuela, a country that has contributed significantly to an increase in imported cases of malaria in this region (BRASIL. Ministério da Saúde, 2020; Arisco et al., 2021). Our findings accordingly underscore the need for constant surveillance, particularly in this area, which has been established to have the highest number of cross-border malaria cases in Brazil.

Despite the importance of monitoring for the presence of parasites with a *pfhrp2* deletion, we showed that the data obtained requires careful analysis. By applying two well-established PCR-based protocols, we found discrepancies in the results obtained regarding the proportions of *pfhrp2* deletion in the same samples. Specifically, whereas the protocol based on conventional PCR indicated a 46% deletion, that based on nested PCR indicated a rate of only 10%. In this context, Parr et al. (2018) recently addressed issues concerning the sensitivity and specificity of PCR assays for *pfhrp2*. The authors demonstrated a tenfold improvement in the limit of detection when the extension temperature of the PCR protocol was lowered from 72°C to 60°C (Parr et al., 2018). In the present study, based on an analysis of three polymorphic microsatellite locus, we showed that most infections were characterized by multiple clones of genetically distinct variants of the parasite. We accordingly speculate that such polyclonal infections may complicate analyses using PCR-based testing methods, given that different parasite populations with and without *pfhrp2* may be present in the same infected individual. For example, polyclonal infections can confound reporting of the true proportion of gene deletions, whereas RDT results may be influenced by the presence of genetically distinct parasites with varying degrees of PfHRP2 expression in the same infection. We detected eight samples with *pfhrp2* deletions and positive results in the RDT; among these, all but one sample harbored polyclonal infections. Preliminary data based on conventional PCR indicated that these samples were *pfhrp3*-negative. However, a method of greater sensitivity needs to be employed to enable a more definitive conclusion regarding *pfhrp3* deletion. Thus, even though this result could be explained in terms of the presence of *pfhrp3*, the diversity of *P. falciparum* populations within the infections could also result in parasite detection by RDTs.

The samples analyzed in this study were collected over a period of 18 years (2002 – 2020), and thus the results obtained provide an indication of how the *pfhrp2* deletion profile has evolved over the years in the Brazilian Amazon. During the assessed period, we detected a moderate and relatively consistent proportion (10%) of *pfhrp2* deletions. In contrast, differing rates of *pfhrp2* deletion have previously been reported in Peru and Colombia. For example, Dorado et al. (2016) recorded a deletion rate of 6% in samples from Colombia collected from different sites between 2003 and 2010, whereas rates reaching up to 18% were detected in samples collected from 1999 to 2009 in a study conducted by Murillo Solano et al. (2015) (Dorado et al., 2012; Murillo Solano et al., 2015; Dorado et al., 2016). Similarly, in Peru, two studies reported distinct deletion rates for samples collected at different sites approximately 5-years apart, with Gamboa et al. (2010) reporting a 41% of deletion (samples collected between 2003 and 2007), whereas Maltha et al. (2012) detected 26% *pfhrp2*-negative isolates (2010 and 2011). Continuous surveillance will provide a clearer picture of how the proportions of gene deletions change over time and across regions.

Although the sequences of *pfhrp2* tend to show considerable diversity worldwide, this variability does not appear to have a substantial influence on the performance of RDTs (Baker et al., 2010; Kumar et al., 2012; Atroosh et al., 2015; Willie et al., 2018). There is, however, evidence to indicate that polymorphisms in the amino acid sequence of PfHRP2 may affect RDT sensitivity at very low parasite densities (<200 parasites/μL) (Baker et al., 2010). To better characterize the variability of PfHRP2, we sequenced the genes of *P. falciparum* isolates representative of the four regions assessed in the present study. As has previously been reported for isolates from other countries (Baker et al., 2005; Baker et al., 2010), we found that all sequences started with a type 1 repeat, ended with a type 12 repeat, and comprised mainly type 2, 6, and 7 repeats. Interestingly, in this regard, a significant relationship has been described between the number of type 2 and 7 repeats and RDT sensitivity at low parasitemia (Baker et al., 2005; Kumar et al., 2012). Nonetheless, there is a yet no consensus regarding this association, and it remains to be established how PfHRP2 variability affects RDT performance (Baker et al., 2010; Kumar Bharti et al., 2017).

We identified 10 distinct patterns of the PfHRP2 sequence based on the type and number of histidine/alanine repeat units present in each sequence, which were mostly exclusive of each region. The only exception in this regard was the pattern III, which was observed at a high frequency in isolates from Rondônia and Mato Grosso collected during the same period. Notably, however, many samples collected in Mato Grosso were from individuals who were probably infected in Rondônia. The high genetic diversity of the parasite population from Roraima was associated to a high variability of PfHRP2. In contrast, for samples collected in the other states, there was not a straightforward association between levels of diversity of parasite and PfHRP2. A comparison of the Brazilian PfHRP2 sequences obtained in the present study with those identified in other South American countries revealed that sequences with similar profiles have been detected in Brazil and French Guiana, particularly the three patterns (I, V, and VI) found predominantly in Roraima and Amapá states. The northeast borders of Brazil that include these two states comprise the “Guiana shield”, along with Suriname, Guyana, French Guiana, and parts of Colombia and Venezuela. This region is characterized by large-scale movements of illegal gold miners and a large number of cross-border malaria cases (Ferreira and Castro, 2019; Arisco et al., 2021). Hence, we might expect to detect extensive gene flow and common patterns of PfHRP2 sequences.

In conclusion, we detected low to moderate levels of *pfhrp2* deletion in different regions of the Brazilian Amazon. Importantly, the presence of *pfhrp2*-negative isolates has not been translated into a reduction in the efficacy of RDTs. We believe that these observations can be attributed, at least in part, to the multiplicity of infection in individuals; although at present, we cannot exclude the possibility of cross-reaction with PfHRP3. We have focused on describing the variability of *pfhrp2* in the Brazilian Amazon, given that PfHRP2 is the main antigen targeted by most RDTs. However, there is evidence to indicate that some monoclonal antibodies used in PfHRP2-based RDTs show cross-reactivity to PfHRP2 and PfHRP3 (Lee et al., 2006). In Brazil, similar to the findings reported from other South American countries (40%–70% of *pfhrp3* deletion), the proportion of *pfhrp3* deletions is typically very high (35%–98%) (Gamboa et al., 2010; Murillo Solano et al., 2015; Dorado et al., 2016; Rachid Viana et al., 2017; Góes et al., 2021). One of the limitations of the present study is that we were unable to assess PfHRP2 levels or the performance of a second brand of RDT to confirm initial results indicating a lack of the protein. Nevertheless, an important finding in this respect was the discrepancy we identified in the proportions of *pfhrp2* deletion detected using two different PCR protocols, which reinforces the need to pay particular attention to laboratory workflows when seeking to assess gene deletions. Taken together, our findings highlight the importance of continuously monitoring the presence and spread of parasites with a *pfrhp2* deletion in the Amazon region to ensure optimal RDT performance.

## Data Availability Statement

The datasets presented in this study can be found in online repositories. The names of the repository/repositories and accession number(s) can be found below: GenBank MZ773486, MZ773487, MZ773488, MZ773489, MZ773490, MZ773491, MZ773492, MZ773493, MZ824674, MZ824675, MZ824676, MZ824677, MZ824678, MZ824679, MZ824680, MZ824681, MZ824682, MZ824683, MZ824684, MZ824685, MZ824686, MZ824687, MZ824688, MZ824689, MZ824690, MZ824691, MZ824692, MZ824693, MZ824694, MZ824695, MZ824696, MZ824697, MZ824698, MZ824699.

## Ethics Statement

The ethical and methodological aspects of this study were approved by the Ethical Committee of Research on Human Beings of the René Rachou Institute (N° 2.243.058), according to the Brazilian National Council of Health (Resolutions 196/96 and 466/ 12). All adult participants signed a written informed consent, whereas next of kin, caretakers, or guardians signed on the behalf of the minors/children enrolled in the study. All the methods were carried out in accordance with the approved guidelines.

## Author Contributions

TS and CJF were the principal investigators. MM, TM, and LF performed the PCR analysis. GC and MM performed sequencing and genotyping analysis. TS, CJF, GC, JL, DP, and AA helped carry out the field work. TS, CJF, CFA, LC, DP, AA, JL, and GC participated in interpretation of the data and critical revisions of the manuscript. All authors contributed to the article and approved the submitted version.

## Funding

This study was funded by the Royal Society of Tropical Medicine and Hygiene (RSTMH Small Grants 2016), Fundação de Amparo à Pesquisa do Estado de Minas Gerais (FAPEMIG), Conselho Nacional de Desenvolvimento Científico e Tecnológico (CNPq), Programa para Inserção de Recém-Doutores nos Programas de Pós-Graduação da Fiocruz and the Programa PrInt-Fiocruz-CAPES. TS, CJF, CFA, and LC are CNPq Research Productivity fellows. GLC thanks the FAPEMIG for scholarship support. The funders had no role in study design, data collection and interpretation, or the decision to submit the work for publication. This study was partially supported by the Coordination for the Improvement of Higher Education Personnel (Coordenação de Aperfeiçoamento de Pessoal de Nível Superior - CAPES) - Finance Code 001.

## Conflict of Interest

The authors declare that the research was conducted in the absence of any commercial or financial relationships that could be construed as a potential conflict of interest.

## Publisher’s Note

All claims expressed in this article are solely those of the authors and do not necessarily represent those of their affiliated organizations, or those of the publisher, the editors and the reviewers. Any product that may be evaluated in this article, or claim that may be made by its manufacturer, is not guaranteed or endorsed by the publisher.
